# Screening in Trauma for Opioid Misuse Prevention (STOMP): study protocol for the development of an opioid risk screening tool for victims of injury

**DOI:** 10.1186/s13722-017-0097-6

**Published:** 2017-12-04

**Authors:** Randall Brown, Brienna Deyo, Chelsea Riley, Andrew Quanbeck, Joseph E. Glass, Rebecca Turpin, Scott Hetzel, Christopher Nicholas, Maireni Cruz, Suresh Agarwal

**Affiliations:** 1 Department of Family Medicine and Community Health, University of Wisconsin, 1100 Delaplaine Ct, Madison, WI 53715 USA; 2 Georgia Department of Public Health, 2 Peachtree Street, NW, 15th Floor, Atlanta, GA 30303 USA; 3 Group Health Research Institute, 1730 Minor Ave #1600, Seattle, WA 98101 USA; 4 National Safety Council, 1121 Spring Lake Dr., Itasca, IL 60143 USA; 5 Department of Biostatistics and Medical Informatics, University of Wisconsin, 600 Highland Ave K6/420, Madison, WI 53792 USA; 6 Hospital Trauma Surgery, University of Wisconsin, 600 Highland Ave G5/335, Madison, WI 53792 USA

**Keywords:** Trauma, Injury, Opioids, Opiates, Opioid addiction, Opioid use disorder, Opioid misuse, Opioid abuse, Screening, Risk factors

## Abstract

**Background:**

Opioid addiction and overdose are epidemic in the U.S. Victims of traumatic injury are at greater than average risk for opioid misuse and related complications. Potential risk screens and preventive interventions in this clinical population remain under-investigated. The current project seeks to develop and pilot the implementation of a screening tool for opioid risk at American College of Surgeons (ACS) Level I and Level II trauma centers.

**Methods:**

The project began with an online survey, which was sent to Wisconsin trauma center medical directors and trauma coordinators for the purpose of gathering information on current substance use screening practices. Next, a focus group of trauma center staff was convened to discuss barriers and facilitators to screening, resources available and needed to support trauma patients with opioid use disorders, and measurable clinical observations that could indicate a patient’s potential risk for opioid misuse. Data from the surveys and focus group were combined to inform the data collection instruments that are currently being administered to patients recruited from the University of Wisconsin Hospital Trauma Inpatient and Orthopedic Surgery Services. Eligible and consenting patients complete standardized measures of socio-demographics, substance use history, opioid misuse risk, mental health, medical history, and injury and pain severity. Follow up visits at weeks 4, 12, and 24 after hospital discharge assess hypothesized risk factors for opioid addiction and opioid use disorder diagnosis. At the completion of patient data collection, a forward stepwise regression will identify factors of most significant risk of the development of opioid use disorder after traumatic injury. This modeling will inform the development of a novel opioid risk screening tool, which will undergo pilot implementation at 4 Wisconsin ACS Level I and Level II trauma centers, using an evidence-based implementation strategy with roots in systems engineering.

**Discussion:**

Positive findings from the proposed work would lead to improved, standardized opioid risk screening practices among victims of traumatic injury. The ultimate goal of this and future work is to reduce the likelihood of opioid misuse, addiction, and related complications, such as overdose and death.

*Trial registration* Clinicaltrials.gov registration number: NCT02861976. Date of registration: Feb 9, 2016

**Electronic supplementary material:**

The online version of this article (10.1186/s13722-017-0097-6) contains supplementary material, which is available to authorized users.

## Background

Opioid use disorders and opioid overdose have reached epidemic proportions in the United States. Effective means to identify risk for opioid-related complications and interventions to prevent opioid use disorders and overdose deaths are, therefore, urgently needed. Nationally, approximately 1.5 million people aged 12 years and older received specialty treatment for heroin or other opioid use disorders in 2015 [1]. Between 2002 and 2011, admissions to substance-abuse treatment programs for prescription opioid use disorder rose by over 400% [2]. Yet, despite the rising prevalence of treatment, there were a record 20,101 deaths due to prescription pain reliever overdose in 2015, and an additional 12,990 deaths due to heroin overdose [3]. In Wisconsin (the geographic setting for the current project), treatment admissions for alcohol use disorder have remained relatively steady over the previous 10 years, while treatment admissions for heroin or other opioid use disorders increased more than 250%. The rate of treatment admissions for heroin or other opioid use disorder surpassed admissions for marijuana use disorder in 2008, and has since remained the most common illicit substance for which treatment is accessed in the state [4]. Yet again, despite the rising prevalence of treatment admissions, overdose deaths from heroin alone have increased more than 600% in Wisconsin since 2005.

Victims of traumatic injury are at greater than average risk for opioid use disorders and, therefore, represent a population with particular need for appropriate assessment, prevention, and intervention protocols; an area which remains severely under-studied. In one study of patients with a recent emergency room visit from which they were discharged with an opioid prescription, rates of misuse were 42% at either 3 or 30 days follow up [5]. In this study, 92% of the participants who reported misuse, reported self-escalation of the prescribed dose. Another study found that the rate of pre-injury opioid use for trauma patients is 16%, compared to 9% in the general population, indicating that pre-injury opioid use may be predictive of post-injury misuse [6].

Pain severity (and associated psychological distress) and pre-injury opioid use have been predictive of a greater duration of post-trauma opioid use [6, 7]. However, physical dependence and behaviors potentially indicative of a use disorder were not assessed in these investigations. Adolescent and young-adult initiates of opioid misuse who progress to injection opioid use frequently cite prescription of an opioid for an injury as their first exposure to opioid, and the event that led to their eventual addiction [8]. Alcohol is also a significant and well-recognized contributor to traumatic injury nationwide. Alcohol misuse predicted opioid misuse in a study of chronic pain patients [9], and was associated with nearly double the rate of opioid misuse (5.2% in low-risk drinkers compared to 9.8% in at-risk drinkers) in a sample of patients with traumatic injuries [7].

Despite this existing knowledge of some of the risk factors for opioid misuse, screening for opioid-related risk is not common practice at trauma centers, and systematic studies examining opioid use disorder screening and prevention in trauma populations have not been undertaken. Given the severity of the opioid addiction and overdose epidemic, such studies are urgently needed.

This study begins the crucial work of preventing opioid use disorder and overdose by developing and pilot testing a novel opioid risk screening tool and protocol. The long-term goal of this and future work is to provide trauma centers with the tools to both identify and prevent opioid misuse by disrupting the progression from prescribed opioid use to opioid misuse to opioid use disorder and related complications, such as overdose and death. Creating a screening tool for OUD risk identification is a necessary first step before designing processes to prevent and intervene with OUD risk in trauma settings, which we intend to do in future research. This work targets the population of traumatic injury victims because: (1) traumatic injury is a sentinel event that predicts subsequent opioid use, which simplifies identification of a group of at-risk individuals; (2) rates of opioid misuse and use disorder are twice as prevalent in post-trauma populations relative to the general population; and (3) opioid risk assessment is not systematically conducted on trauma services, and the feasibility and effectiveness of such a protocol remains under-investigated.

## Methods

### Primary aims

#### Aim 1

In a sample of traumatic injury victims (n = 295) at an American College of Surgeons, Level I trauma center, collect data on hypothesized risk factors for opioid misuse after traumatic injury, and monitor for the development of opioid use disorder during the 24 weeks follow up period.

#### Aim 2

Using the variables collected in Aim 1, develop a multivariate regression model of the proximal indicators that are most strongly associated with the development of opioid misuse or opioid use disorder (a composite outcome) after traumatic injury. Then, use this model to develop a parsimonious set of opioid risk screening items into a novel opioid risk screening tool which can be feasibly implemented at trauma centers.

#### Aim 3

Pilot test the implementation of the opioid risk screening tool at ACS Level I and II trauma centers in Wisconsin.

### Outline of data collection

To address Aim 1, preliminary data collection was necessary to inform the specific instruments and supplementary questions that would be administered to traumatic injury patients. First, an online survey was sent to Wisconsin trauma center medical directors and trauma coordinators to gather information on current substance use screening practices. Details regarding the procedures and results of these activities are attached in Additional file 1: Online Supplement 1. Next, a focus group of Wisconsin trauma center staff was convened to discuss barriers and facilitators to screening, resources available and needed to support trauma patients at risk for opioid use disorders, and measurable clinical observations that could indicate a patient’s potential risk for opioid misuse. Details regarding these procedures and results are attached in Additional file 2: Online Supplement 2. Finally, these data were used to finalize the items that are currently being administered to inpatients receiving trauma services (n = 295) at an ACS Level-1 trauma center in Wisconsin. In turn, these data will be analyzed and used for the development and validation of a screening tool for opioid use disorder risk after traumatic injury. The screening tool will be pilot-tested at four ACA Level-I and Level 2 II trauma centers in Wisconsin.

### Data collection from patients with traumatic injury

#### Sample, eligibility, and recruitment

Participants (n = 295) will be recruited from the UW Hospital Trauma Inpatient Service, which has an annual volume of 2000–3000 patients. Inclusion criteria are age 18–75 years, inpatient victim of traumatic injury, expected need for post-discharge outpatient opioid analgesia, English speaking, and post-discharge ability of participants to manage their own medications. Exclusion criteria are disposition to a skilled nursing or long-term acute care facility where medications are managed by people other than the participant, active opioid use disorder or current participation in a program of recovery for another substance use disorder, current cancer diagnosis, and inability to consent to research due to incapacitating injury or sedation. The electronic medical record (EMR) will be used for pre-screening Trauma Service inpatients for study eligibility. Potentially eligible patients will be offered information about the study. If clinical care staff confirms that the patient is able to provide consent for research and the patient is interested, they will complete an Informed Consent process. Final eligibility criteria will be assessed, including a computer-assisted diagnostic survey at baseline (the Composite International Diagnostic Interview-Substance Abuse Module) [10] to assess for existing opioid use disorder. Eligible patients will be enrolled in the study.

#### Participant surveys

Baseline visits will be completed during the inpatient stay or within 1 week of discharge, with follow-up visits at weeks 4, 12, and 24 after hospital discharge. Surveys will assess sociodemographic characteristics and health characteristics hypothesized to predict opioid use disorder risk that are not available in the EMR. The baseline and 24-week survey must be completed in person. The 4 and 12-week follow-up surveys were designed to be completed via mail, in-person, or by telephone. If participants complete all 4 visits, they will be compensated a total of $150, to be disbursed in increments at each visit. All study data will be transcribed from hard-copy questionnaires and source documents into a secure, web-based application for storing, managing, and analyzing study data.

#### Administrative data sources

To assess hypothesized risk factors for opioid misuse or opioid use disorder, informed consent will give the study team permission to record personal health information from administrative records including the EMR, UW Health Trauma Registry, Wisconsin Circuit Court Access Program (CCAP), and Wisconsin PDMP Database. Administrative data sources include: emergency department notes and procedures from the EMR; Injury Severity Score (ISS) from the Trauma Registry [11], diagnosis codes from the EMR and trauma registry; concomitant medications, prescribed opioids, and other methods of pain management from the EMR and PDMP; and criminal activity from the CCAP. These data will be collected at week 24, at the conclusion of participant follow ups.

### Primary outcome

The primary outcome is the development of opioid misuse or opioid use disorder during 24 weeks follow up, as measured by the Composite International Diagnostic Interview-Substance Abuse Module (CIDI-SAM) and/or the current opioid misuse measure (COMM).

The CIDI-SAM is a computer-assisted interview (average test–retest reliability *Κ* = 0.84) that identifies opioid use disorder presence and severity, based on symptom clusters, according to the 5th Edition of the Diagnostic and Statistical Manual of Mental Disorders. [10, 12, 13] The COMM is a 17-item paper survey with 5-point Likert scale response options, which will be used to identify the presence and severity opioid misuse behaviors. In order to determine changes in these behaviors over time, COMM will be administered at each follow up. The COMM has acceptable one-week test–retest reliability and internal validity (Intra-class Correlation Coefficient [ICC] = 0.86; 95% CI 0.77–0.92) [14, 15]. A raw score of greater than or equal to 9 will be considered positive for opioid misuse.

The composite outcome will be positive for participants with positive scores at week 24 on either the CIDI-SAM (opioid use disorder) or the COMM (opioid misuse), and will be negative for participations with neither.

### Independent variables

A number of independent variables will be collected throughout the 24 weeks follow up. The inclusion of these measures are based on evidence from literature, and the surveys and interviews with Wisconsin trauma center staff and coordinators that were conducted as an earlier part of this study. The follow up time points were determined by the need to regularly evaluate for ongoing opioid use.

The baseline measures will be assessed for the development of a screening tool that could be administered at the point of care for a traumatic injury. Measures administered at follow up time points will be assessed for the development of a screening tool that could be administered by trauma staff at post-discharge follow up appointments or by primary care after a handoff from a trauma service to a clinical service.

Sociodemographic information (including age, sex, race, place of birth, work status, marital status, and household income and inhabitants) are collected at baseline. Additional baseline measures include: depression screen (PHQ-9); anxiety screen (GAD-7); Opioid Risk Tool (ORT); Social Support Questionnaire (ISEL-12); description of mechanism of injury; whether or not the injury was work related; number of procedures and/or complications associated with the injury; medication allergies; and time spent on an intensive care unit.

Ongoing opioid use after hospital discharge is recorded at each visit in order to establish a Morphine Equivalent Daily Dose (MEDD) per time point, as well as to determine the date of last opioid use.

The Pain Medication Questionnaire (PMQ) [16, 17] is a 23-item instrument assessing prescription use behaviors, whose score is associated with physician assessments of opioid medication risk. The instrument has excellent test–retest reliability (coefficient of stability 0.086); and acceptable internal consistency (Cronbach’s alpha = 0.73). This instrument is collected at each visit in an attempt to determine the optimal time for administering an opioid risk screening tool.

The Post-Traumatic Stress Disorder (PTSD) Check List (PCL-5) is a 20-item checklist that measures symptoms on a 5-point Likert scale. The instrument provides an overall score, and domain scores based on symptom clusters. The PCL-5 is used once at baseline, to evaluate for PTSD symptoms that may have existed at the time of the injury, and again at week 12 to evaluate specifically for PTSD symptoms that may have developed and persisted as a result of the event that caused the traumatic injury.

The Pain Catastrophizing Scale (PCS) [18, 19] asks participants to indicate the intensity of each of 13 thoughts or feelings associated with past pain episodes on 5-point scales. The PCS yields a total score and three subscale scores assessing rumination, magnification and helplessness. The PCS has been shown to have adequate to excellent internal consistency (coefficient alphas: total PCS = 0.87, rumination = 0.87, magnification = 0.66, and helplessness = 0.78). The PCS is administered at baseline to assess pain catastrophizing as close to the time of the injury as possible, and again at week 24 to assess for changes that might occur to pain catastrophizing one the participant is no longer in acute pain.

In addition to the CIDI-SAM, which constitutes the primary outcome, the Alcohol Use Disorders Identification Test (AUDIT-C) [20] will be collected at both baseline and week 24. The AUDIT-C consists of 3 items that are validated to assist in identifying hazardous alcohol consumption patterns.

The Adverse Childhood Experience (ACE) Questionnaire consists of 10 yes/no items that describe scenarios, including childhood abuse, neglect, and household dysfunction. These types of adverse childhood experiences have been strongly correlated with substance misuse and use disorder in adulthood. [21] Since all of the participants in this study are adults, this instrument is administered at week 4, rather than baseline, in an effort to reduce survey fatigue at baseline.

The Brief COPE Inventory (COPE), the Opioid Craving Scale (OCS), the Patient-Reported Outcomes Measurement Information System (PROMIS) Global Health Scale, Pain Intensity and Interference (PEG), and the Perceived Need Inventory (PNI) are all administered at each follow up time point in an attempt to capture changes in various areas that may correlate with opioid tapering and discontinuation. The COPE is a 26-item, 4-point Likert scale survey that measures the type and frequency of coping strategies in 13 different domains, including substance use, religion, emotional support, positive reframing, disengaging, humor, self-blame, etc. The OCS consists of three visual analog scales that measure current craving, craving in risky situations, likelihood of giving into craving. The OCS is a composite measure of craving, which exhibits good internal consistency (Spearman’s rho range from 0.85 to 0.92) and predictive validity of future opiate use [22]. The PROMIS is a 10-item measure of general quality of life, which includes a physical health domain (Cronbach’s α  =  0.81), and a mental health domain (Cronbach’s α  =  0.86) [23]. The PEG and PNI both use 11-item Likert scales. The PEG is a 3-item instrument that measures level of pain, and how that pain interacts with enjoyment of life and general activity. The PNI is a 5-item instrument that measures perceived problems with opioid use, including the need for opioid use treatment and the perceived potential effectiveness of that treatment [24–27].

At the conclusion of the follow up period, data will be collected about each participant’s felony and misdemeanor record, and their prescription drug activity. Felony and misdemeanor records will be obtained from public court records from the Wisconsin Circuit Court Access Program (CCAP). A limitation to CCAP data is that it is only available for offenses that occurred within the state of Wisconsin. Prescription drug activity will be collected from the Wisconsin electronic Prescription Drug Monitoring Database (PDMP). Data on prescription opioid fills can be obtained from Wisconsin and 14 other states (Table 1).

**Table 1:** Aim 1 measures and follow-up timeline

Measure title	Baseline	Week 4	Week 12	Week 24
Sociodemographic characteristics	X			
Morphine Equivalent Daily Dose (MEDD)	X	X	X	X
Pain Medication Questionnaire (PMQ)	X	X	X	X
Post-Traumatic Stress Disorder Check List (PCL-5)	X		X	
Pain Catastrophizing Scale (PCS)	X			X
Alcohol Use Disorders Identification Test (AUDIT-C)	X			X
Composite International Diagnostic Interview-Substance Abuse Module for Opioid Use Disorder (CIDI-SAM)	X			X
Patient Health Questionnaire (PHQ-9)	X			
Generalized Anxiety Disorder (GAD-7)	X			
Opioid Risk Tool (ORT)	X			
Social Support Questionnaire (ISEL-12)	X			
Adverse Childhood Experiences (ACE)		X		
Brief COPE Inventory (COPE)		X		X
Current Opioid Misuse Measure (COMM)		X	X	X
Opioid Craving Scale (OCS)		X	X	X
Quality of Life Questionnaire (PROMIS)		X	X	X
Perceived Need Inventory (PNI)		X	X	X
Pain Intensity and Interference (PEG)		X	X	X
WI Circuit Court Access Program (CCAP)				X
WI Prescription Drug Monitoring Database (PDMP)				X

### Pilot implementation of the risk screening tool

Study Aim 3 relies upon a well-established approach to the implementation of evidence-based practices, based on fundamental principles of organizational change developed by the Network for the Improvement of Addiction Treatment (NIATx) [28–30]. Of the approaches used to deliver the NIATx organizational change model, peer coaching has emerged as the most cost-effective option [28]. Members of the research team have adapted the peer coaching model employed by NIATx and applied it to promoting adoption of clinical guidelines for opioid prescribing in primary care [31]. Further adaptations will be made to tailor the approach for trauma settings. Peer coaches guide organizations through the change process by assessing workflows and identifying opportunities for improvement, and facilitating the plans, actions, analysis, and adoption of those improvements. This study will culminate with the pilot test of a strategy for implementing an opioid risk screening protocol in Wisconsin’s 4 largest trauma centers, using the novel opioid risk screening that was developed from the trauma patient data.

Implementation work will begin with observations of the UW Hospital Trauma Inpatient Service clinical workflows regarding the Alcohol Use Disorders Identification Test (AUDIT-C), a standardized alcohol use screening tool, a process which will inform potential implementation procedures for the opioid risk screen [32, 33]. This observation period will focus on when, where, and who is administering the AUDIT-C, how the information obtained from the AUDIT-C is recorded and communicated to the clinical care team, if and how that information guides clinical care decisions, and if and how an intervention or referral to treatment is conducted with the patient. Interviews will be conducted with UW Hospital Trauma Inpatient Service staff to assess how opioid risk screening could fit within the trauma inpatient workflow and how opioid-related interventions might best dovetail with that workflow. Nuances of the implementation model that are specific to a trauma service will be shared from the UW site to the four other pilot testing sites. The implementation model will be continuously refined based on the experience of each subsequent implementation, with emphasis placed on adjustments that are needed to tailor the implementation model for local organizational contexts.

### Statistical analyses

#### Aims 1 and 2: sample size calculations

Based on an expectation of an at-least 8% incidence in the sample over 24 weeks observation of opioid misuse or an opioid use disorder diagnosis, 221 participants would yield 80% power, at a 5% significance level, with a logistic regression to detect an odds ratio of 2.0 for a change of one standard deviation (SD) in score on the COMM. With a 25% drop-out rate expected, the study aims to recruit a total of 295 participants. While the current proposed work will not be adequately powered to attain significant findings regarding predictors of opioid overdose, examination of this outcome will constitute an exploratory aim.

#### Aims 1 and 2: analysis

The primary outcome event of this study will be a composite outcome indicating the development or lack of opioid misuse or opioid use disorder within 24 weeks of hospital discharge after traumatic injury. To assess the associations between patient factors and hypothesized risk factors with primary event, t-tests, Wilcoxon rank sum tests, and Chi square tests will be applied to measures that differ between those participants who achieve the primary event and those who do not. The test that is used will be based on numeric (normal or non-normal distribution) or a categorical factor. Any baseline or week 4 variable that is univariately associated with the primary event (p < 0.05) and does not reduce the number of events by more than 5% due to missing data will be considered a candidate variable to be included in the development of a best fit prediction model. The prediction model will be constructed based on a forward step-wise logistic regression model building process with threshold for entry into the model of significantly improving the model at a 0.05 significance level. Including baseline and week 4 variables will allow knowledge of which baseline variables are associated with future opioid misuse or opioid use disorder, and which variables can indicate the beginning of addiction in the hopes to develop a method to intervene before the onset of opioid misuse or opioid use disorder. To control for a possible early drug effect, time-on-opioids will be used as a covariate in the model. The diagnostic ability of the model will be assessed by constructing a receiver operating characteristic (ROC) curve and calculating the optimal sensitivity and specificity to predict primary event based on Youden’s J statistic [34]. A secondary survival analysis will examine variables that are associated with time-to-event, such as overdose or incident misuse detected on study measures.

#### Aim 3: analysis

Aim 3 analysis will involve mixed methods. Formative evaluation will inform the implementation model, based on pre-implementation stakeholder interviews. Quantitative evaluation of the implementation process is based on the Reach, Effectiveness, Adoption, Implementation, Maintenance (RE-AIM) model [35]. The following outcomes will be assessed pre-post implementation in pilot test sites: (1) Reach: Number and characteristics of patients served in pilot trauma centers compared to patients of trauma centers nationally; (2) Effectiveness: number and percentage of trauma patients screened and receiving follow-up; (3) Adoption: number and characteristics of providers conducting screening/follow up with patients; (4) Implementation: number and characteristics of providers participating in the coaching intervention; intervention “dose” received; and adaptions made to the implementation model for each site. Assessing the dose of intervention received will be defined as the number of coaching hours delivered to clinic staff, and tracked by coach logs. To measure adherence, the planned protocol will be reviewed with clinicians and site staff, and adaptations made to the protocol at each site will be documented and reviewed. Quality of the intervention delivery will be assessed by asking clinicians to reflect on their experience with peer coaching, and the effect it had on their attitudes about opioid prescribing. Providers and hospital staff who were involved with peer coaching will be invited to participate in a focus group, which will explore the kinds of workflow changes that were associated with successful implementation, what factors helped staff make changes, what factors acted as barriers to change and how those barriers were addressed, and what aspects of implementation did not work well. Focus groups will also seek to compare the experience of clinicians at sites that successfully implemented the opioid screening protocol into their workflow compared to those at sites that did not.

## Discussion

The primary anticipation is that a parsimonious set of injury-related and historical factors will predict opioid misuse and the development of opioid use disorders during the period of observation and that these screening items are feasibly implemented during the course of the management of the injured patient. While it is not expected that these results will achieve adequate power to indicate statistically significant findings related to opioid overdose and mortality, collected patient factors may demonstrate trends toward predictive value for these outcome as well, which might be further developed in future study. The full project will develop 3 products: (1) a screening tool that describes patient opioid-related risk; (2) an implementation strategy for integrating the developed screening instrument into standard clinical care, and (3) preliminary strategies for interventions to prevent opioid misuse, overdose and addiction to be tested in future study, which will be informed by the AUDIT-C workflow analysis and by opioid-related risk factors identified by traumatic injury patient surveys.

While the primary purpose of the current work is the development of an effective opioid misuse screening tool, an important consideration during the course of the work will be imagining the form that an intervention aimed at preventing opioid misuse might take in future research. This intervention could manifest in a variety of forms, including something similar to ‘brief interventions.’ However, an effective intervention also might relate to effective management of pain, and/or appropriate prescribing and monitoring.

An additional question becomes “at what point should results of screening begin to affect care?” Clearly, appropriate and effective pain management is a primary concern during the acute hospital stay. Effective pain management reduces the likelihood of potentially catastrophic complications, such as pneumonia, heart attack, pulmonary embolism, impaired healing, and injury-related post-traumatic stress disorder [36].

How opioid prescribing might be modified for traumatic injury care in order to reduce the risk of opioid misuse is unclear. Perhaps some cues might be taken from chronic opioid prescribing guidelines, such as those put forward by the Centers for Disease Control and Prevention in 2016, whose recommendations include prescription drug monitoring database surveillance and periodic random urine drug testing of patients with opioid prescriptions. Checks of state prescription drug monitoring programs could provide valuable information regarding the frequency of refills and utilization of emergency departments for pain care after discharge, provided those systems are adequately updated and maintained; however, the utility of urine drug testing in this clinical situation has not yet been examined. The use of treatment agreements and urine drug screening is becoming increasingly commonplace in primary care. These two metrics were the primary measures of guideline concordant care used in a recent evaluation of a nurse case management intervention for opioid patients in primary care [37]. While these safety measures may not be wholly sufficient to prevent addiction and misuse in all cases, an environmental scan conducted at baseline indicated that urine drug screening, prescription drug monitoring program checks, and treatment agreements are rarely (if ever) used in the trauma centers participating in the pilot implementation portion of this study. Instituting these universal precautions would thus be a positive first step towards building safer systems for opioid prescribing in trauma centers. Trauma centers will be encouraged to exercise these precautions for patients who screen positive for potential misuse.

Ultimately, the current study and future work seeks to facilitate improved clinical care as it relates to opioid risk reduction while ensuring appropriate pain management for victims of traumatic injury. If successful, this work might also provide a screening tool applicable to other clinical settings for pain management and opioid risk reduction.

## Additional files

**Additional file 1** STOMP Trauma Center Survey.

**Additional file 2** STOMP Focus Group with Trauma Center Staff.

## Abbreviations

ACE: adverse childhood events
ACS: American College of Surgeons
BAC: blood alcohol concentration
CCAP: Circuit Court Access Program
CIDI-SAM: Composite International Diagnostic Interview-Substance Abuse Module
COMM: current opioid misuse measure
COPE: brief Coping Inventory
EMR: electronic Medical Record
GAD-7: Generalized Anxiety Disorder-7 items
ICC: Intra-class Correlation Coefficient
ISEL-12: Interpersonal Support Evaluation List-12 items
ISS: Injury Severity Score
MEDD: Morphine Equivalent Daily Dose
NIATx: Network for the Improvement of Addiction Treatment
OCS: Opioid Craving Scale
ORT: Opioid Risk Tool
PCL-5: PTSD Check List-5 items
PCS: Pain Catastrophizing Scale
PDMP: Prescription Drug Monitoring Program
PHQ-9: Patient Health Questionnaire-9 items
PMQ: Pain Medication Questionnaire
PNI: Perceived Need Inventory
PROMIS: Patient-Reported Outcomes Measurement Information System
ROC: Receiver Operating Characteristic
SD: Standard Deviation
STOMP: Screening in Trauma for Opioid Misuse Prevention
UW: University of Wisconsin
