# Supplementary material for: Screening in Trauma for Opioid Misuse Prevention (STOMP): study protocol for the development of an opioid risk screening tool for victims of injury
Source: Addict Sci Clin Pract. 2017 Dec 4;12:28. doi: 10.1186/s13722-017-0097-6 (PMC5713647; doi:10.1186/s13722-017-0097-6)
Supplement: Supplementary file 1 — Additional file 1 STOMP Trauma Center Survey. [file 13722_2017_97_MOESM1_ESM.docx]

Online Supplement 1

STOMP Phase 1 Activities

Title: Online survey of Wisconsin trauma center staff

Rationale for survey: While screening for at-risk and problem alcohol use is widespread (and often required) practice at trauma centers, little is known about the frequency and structure of current screening practices for risk related to other substances, such as prescription opioids. This survey seeks to clarify the form which this practice takes at Wisconsin trauma centers. Additionally, the survey seeks to characterize existing resources which might be leveraged if such screening practices were to be implemented in locations where they currently are not performed.

Methods:

Sampling frame—Contact information for clinical trauma staff in Wisconsin was obtained from the Wisconsin Department of Health Services’ State Trauma Coordinator. Only staff of American College of Surgeons’ verified Level I and II and State of Wisconsin verified Level III and IV trauma centers were contacted for potential participation. This resulted in a list of122 contacts at 57 hospitals in the state.

Data collection procedures—Eligible participants were emailed brief information as to the nature of the survey and a link for survey completion. No identifying participant information was collected. (#) reminder emails were sent in an effort to ensure an adequate response rate. The survey was prepared in Qualtrics^1^ and responses uploaded to a password-protected database.

Instruments—see attached survey.

Results: Responses were received from 21 staff members from 14 institutions. Hospital bed numbers at participant institutions are shown in Figure 1 and trama level verification in Figure 2.


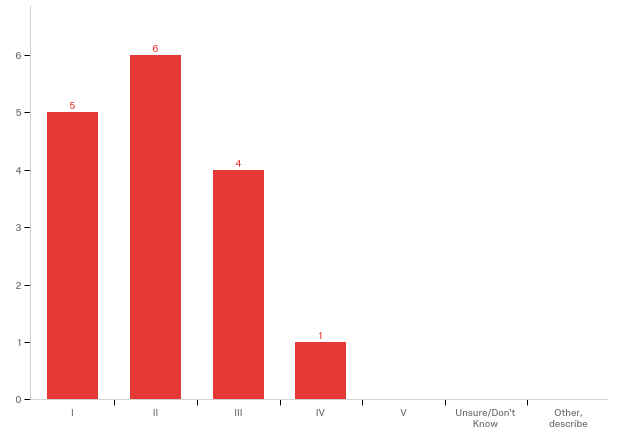


Figure 2. Trauma level of respondent institutions.

Fifty-six percent (n = 9) of those responding to the item (n = 16), stated that their trauma services routinely screened for substance misuse other than alcohol. Eighteen percent (n = 3) responded “no,” 25% (n = 4) “unsure.” Those responding (n = 12) described the screening process as clinical interview (67%, n = 8), urine testing (58%, n = 7), or standardized questionnaire (25%, n = 3). One respondent was “unsure.”

When asked about opioid risk screening in particular, 71% (n = 10) of those responding (n = 14) stated this was not routine practice, 14% (n = 2) endorsed this as routine practice, and 10% (n = 2) were unsure. For the institutions endorsing opioid risk screening as a routine practice (n=6), two were unsure of what method was used; two cited “clinical interview;” one cited “standardized questionaires;” and one cited testing of blood/urine. The respondent indicating that a standardized questionnaire was used did not identify an instrument, but, rather, indicated that an RN questioned patient at the time of admission. When asked about available interventions for patients screening positive for opioid-related risk, respondents described the following resources: brief intervention in the hospital (n=2), referral to internal AODA service provider (n=2), referral to community-based AODA treatment (n=2), and unsure (n = 2).

Discussion: The survey sought to provide initial descriptive information on current practices related to substance use screening at Wisconsin Trauma Centers, with a focus on opioid misuse. The results in this small sample indicated that routine screening rates for opioid misuse are likely quite low and that standardized processes are likely even more rare. While quite limited in size and scope, these data provided a fertile basis for the initiation of discussion at a Trauma & Opioids Summit that was conducted as part of the overall study. Results of the provider focus group which took place at that Summit are attached as Appendix B.

1. *Qualtrics*. (Qualtrics, 2017).

**Trauma patients and opioid misuse risk survey**

While trauma patients may present increased risk for problem opioid use, neither standard screening processes nor potential interventions to prevent opioid misuse and addiction have been systematically studied in trauma populations. We are interested in collecting information from American Trauma Centers about current practices and barriers to their expansion in an effort to develop efficient tools for identifying opioid misuse and addiction risk. Thank you, once again, for your time and attention.

**Hospital Characteristics:**

1. How many beds are in your Trauma Center?
2. On average, how many trauma admissions does your Trauma Center have annually?

<500 2501-3500

501-1000 3501-4500

1001-1500 4501+

1501-2500

1. Is your hospital an ACS/State verified Adult Trauma Center? Yes No
   1. If yes, what level verification is your Adult Trauma Center I II III IV V
2. Is your hospital an ACS/State verified Pediatric Trauma Center? Yes No
   1. If yes, what level verification is your Pediatric Trauma Center I II III IV V
3. Does your Trauma Center have a surgical residency training program? Yes No

**Current screening practices**:

1. What instrument does your Trauma Center currently use to screen for at-risk and problem alcohol use in trauma patients?

(Mark all that apply)

- 1. CAGE
  2. AUDIT
  3. AUDIT-C
  4. Consumption Questions
  5. Interview
  6. None
  7. Other (explain)________________

1. In what way does your institution intervene for patients who screen positive for at-risk or problem alcohol use? Check all that apply
   1. Brief Intervention,
   2. Referral to internal Alcohol and Other Drug Abuse (AODA) provider,
   3. Referral to community AODA,
   4. No Intervention
   5. Other (explain)___________
2. What instrument does your institution currently use to screen for **opioid** misuse and addiction risk in trauma patients?
   1. Standardized instrument (please identify)___________________________,
   2. no current screening
   3. other (explain)_____________________________

Please indicate your agreement with the following statements on a scale from 1 (strongly disagree) to 5 (strongly agree).

|  | Strongly Disagree |  | Neutral |  | Strongly Agree |
| --- | --- | --- | --- | --- | --- |
| 9) "Opioid misuse and addiction are critical problems in the United States." | 1 | 2 | 3 | 4 | 5 |
| 10) “Screening for opioid misuse and addiction risk is clinically valuable." | 1 | 2 | 3 | 4 | 5 |
| 11) "Early intervention would prevent progression from opioid misuse to addiction." | 1 | 2 | 3 | 4 | 5 |

12) Please rank the following barriers to screening for opioid misuse and addiction risk in your Trauma Center, with 1 being not a barrier and 5 being a major barrier.

|  | Not a Barrier |  | Somewhat of a barrier |  | Major Barrier |
| --- | --- | --- | --- | --- | --- |
| Lack of evidence to support the practice | 1 | 2 | 3 | 4 | 5 |
| Lack of time for staff to engage in this practice | 1 | 2 | 3 | 4 | 5 |
| Lack of available resources within my institution to follow-up on positive screens | 1 | 2 | 3 | 4 | 5 |
| Lack of available resources within my community to follow-up on positive screens | 1 | 2 | 3 | 4 | 5 |
| Lack of training in addressing addiction and addiction risk | 1 | 2 | 3 | 4 | 5 |
| Lack of a requirement by my accrediting body or institution to perform an opioid misuse risk screening | 1 | 2 | 3 | 4 | 5 |

|  |  |  |  |  |  |
| --- | --- | --- | --- | --- | --- |
| Staff and processes already in place for alcohol screening could serve as a model | Have and Supports opioid screening | Have but may not support opioid screening | Do not Have | Don’t Know |  |
| There is a current system of follow-up for trauma patients prescribed opioids which could assess use and misuse | Have and Supports opioid screening | Have but may not support opioid screening | Do not Have | Don’t Know |  |
| Available process in place for referrals to appropriate consultation which address opioid misuse (substance abuse treatment centers, addiction medicine specialists, pain medicine specialists, etc.) | Have and Supports opioid screening | Have but may not support opioid screening | Do not Have | Don’t Know |  |
|  |  |  |  |  |  |

1. Please identify any other barriers to screening for opioid misuse and addiction risk in your Trauma Center, other than those listed in question 12.
2. Please identify resources within your institution which you believe support opioid risk screening and intervention:
3. Please describe any other resources within your Trauma Program that may support the implementation of opioid misuse risk screening.

*Please provide the following information about the person completing this survey.*

1. What is your role within your Trauma Program? _______________________________________
2. What is the name of your hospital? (for de-duplication purposes only , will be removed in analysis) _____________________________________________________________

Thank you!
