# Supplementary material for: Screening in Trauma for Opioid Misuse Prevention (STOMP): study protocol for the development of an opioid risk screening tool for victims of injury
Source: Addict Sci Clin Pract. 2017 Dec 4;12:28. doi: 10.1186/s13722-017-0097-6 (PMC5713647; doi:10.1186/s13722-017-0097-6)
Supplement: Supplementary file 2 — Additional file 2 STOMP Focus Group with Trauma Center Staff. [file 13722_2017_97_MOESM2_ESM.docx]

Supplement 2: STOMP Phase 2 Activities

Focus Group with Wisconsin Trauma Center Providers and Staff

**Rationale**

A focus group comprised of trauma center providers and staff, was held for the purpose of characterizing: perceptions of patient risk factors for opioid-related complications, characteristics of opioid-risk screening measures that might feasibly be implemented on trauma services, and anticipated logistic issues, such as staffing, for screening/intervention administration. Data from the focus group was used to further inform the Phase 3 patient survey instruments, and the Phase 4 implementation plan.

**Methods**

The focus group was held in person at the conclusion of the Summit on Trauma and Analgesia that was held in Madison, WI on September 9, 2016. Study data was not collected during the Summit itself, but Summit attendees were recruited upon registration to participate in the focus group. The focus group was held after a brief break after the conclusion of the Summit, in the same location as the Summit. Dr. Brown and Dr. Quanbeck co-lead the focus group, while other study team members took notes. The focus group was audio recorded and transcribed for later reference. Team member notes and the audio transcript represent the data set for this phase of the study.

Focus Group Topics:

- Perception of risk factors for opioid-related complications (overuse, addiction, misuse) in your patient population
- Perception of opioid-related risks and challenges in the course of usual clinical care for traumatic injury
- Beliefs regarding potential effectiveness of opioid-risk screening and early intervention
- What modifications or shifts in practice have you or your institution recently made to address opioid misuse?
- How might prescribing/monitoring be modified in the “ideal world” (i.e. if you had all the resources you needed to make it happen?) if a trauma victim is clearly at risk for opioid misuse/addiction?
- What do you envision screening for opioid misuse risk to look like for your institution?
- What resources does your institution currently have that might be brought to bear for purposes of screening/brief intervention?
- What role might there be for hospitals and trauma services in preventing opioid misuse/addiction (or should this fall to primary and other specialist care)?

**Results**

In total, 9 people participated in the focus group, which consisted of RNs, NPs, and/or MDs from 4 Wisconsin hospitals. The focus group lasted approximately 70 minutes. Participants were engaged and respectful, even in disagreement. The fact that all participants were care providers on trauma teams meant that they all came in to the discussion with similar contextual understandings of pain management for traumatic injury patients. Further, participants were able to help each other articulate ideas that stalled with the phrase, “do you know what I mean?” Based on the topics discussed in the focus group, Phase 3 data points were updated.

Phase 3 data collection changes:

- Increased inclusion criterion for age from 65 years to 75 years
- Deleted exclusion criterion for pre-injury chronic opioid use, and clarified that only opioid use that met criteria for misuse would be exclusionary
- Deleted exclusion criterion for active participation in a program of recovery for another substance use disorder, allowing enrollment of people with active use disorders for non-opioids
- Added BPI at hospital admission and discharge to baseline EMR data collection
- Added indication of whether or not the injury was work related to baseline EMR data collection
- Added notes about duration, unit, and ventilator use to baseline EMR data collection
- Added AUDIT-C score at hospital admission to baseline EMR data collection
- Specified use of CIDI-SAM to evaluate for opioid use disorder only, not for other substances
- Added pain medications administered and procedures performed during inpatient stay to baseline EMR data collection
- Added ISEL-12, a standardized measure of social support to baseline visit
- Added the ACE, a standardized measure of childhood trauma to the 4 week follow up
- Added the Brief COPE, a shortened standardized coping scale to the 24 week follow up visit
- Added AUDIT-C, a standardized alcohol risk screening instrument to the 24 week follow up visit

**Discussion**

Although the literature regarding opioid prescribing in the setting of chronic pain provided some guidance as to measures of putative risk factors, the qualitative data collected in a focus group of clinical providers at Wisconsin trauma centers contributed to the development of further data collection and additional potential risk factors of importance.
